# Computing a cure for fragile-X syndrome

**DOI:** 10.1093/braincomms/fcae066

**Published:** 2024-03-05

**Authors:** Teddy Mohamad, Jean-François Lepage

**Affiliations:** Department of Pediatrics, Faculty of Medicine and Health Sciences, Sherbrooke University, Sherbrooke, Canada, J1H 1B9; Sherbrooke University Hospital Research Center, Sherbrooke, Canada, J1H 1B9; Department of Pediatrics, Faculty of Medicine and Health Sciences, Sherbrooke University, Sherbrooke, Canada, J1H 1B9; Sherbrooke University Hospital Research Center, Sherbrooke, Canada, J1H 1B9

We would like to comment on the work of Chadwick *et al*.^1^ reporting the effects of a combined pharmacological intervention in the fragile-X syndrome (FXS) mouse model. FXS results from the absence of the fragile-X protein (FPX), which has pleiotropic effects. As a result, the manifestation of FXS is multifaceted, characterized both by marked alterations in the cognitive (e.g. executive function) and behavioural (e.g. hyperactivity) domains. There is currently no cure for FXS.

In their work, the authors investigated a combination of repurposed drugs to address the phenotypic complexity of FXS, with remarkable results. First, they showed that gaboxadol (a GABAα-receptor agonist) and ibudilast (a phosphodiesterase inhibitor) improved specific aspects of FXS symptomatology when used separately: gaboxadol improved behaviour while ibudilast enhanced cognitive performance in in the mouse model. Building on this, they tested a therapy combining the two molecules, which led to marked improvements in both domains.

While using a polypharmacological approach for FXS is not a new idea in itself^2^—some clinical trials have been conducted^3^—the work of Chadwick and collaborators is nonetheless ground-breaking. The most innovative aspect of their work resides in the way they proceeded to select the drugs to be tested in their model where two complementary computational approaches were used. The first one considered the genes that are abnormally expressed in FXS to identify drugs that may reverse their mis-regulation, whereas a second identified the combination of drugs offering maximal coverage for the targets of interest. This data-driven technique contrasts markedly with previous endeavours testing polypharmacology in FXS, which were based solely on theoretical grounds^4-6^. In doing so, Chadwick and colleagues elegantly handled the caveats associated with selecting amongst the thousands of mRNA targets of FXP, and defined a combination of drugs that is parsimonious and potent.

The impacts of this drug combination on FXS symptomatology were investigated in a flawless design, in which a combination of dosages and drug regimens was assessed. Importantly, and setting them apart from several previous endeavours, the authors tested the effect of chronic dosing, an important aspect for eventual clinical use as drug tolerance may be associated with recent unsuccessful clinical trials.^7^ In that regard, the FXS community is all too familiar with the translational gap between animal models and human patients, which is partly related to the inadequacies of current outcome measures. As highlighted by the Fragile-X Outcome Measures Working Group,^8^ ‘There is a critical need for objective direct measures of [central nervous system] function that can provide feedback about a drug’s engagement to its target.’ While assessing the direct effects of gaboxadol and ibudilast on the human brain *in vivo* is challenging, transcranial magnetic stimulation (TMS) may provide significant clues into therapeutic target engagement considering its proven sensitivity to the modulatory effects of gabaergic drugs and its capacity to track changes in excitability.^9^ In this respect, recent work from our group has shown the feasibility and relevance of using TMS in the context of clinical studies in FXS.^10^ Future work combining techniques that provide mechanistic insights into the effects of drugs on the brain, with novel computational approaches as those put forth by Chadwick and colleagues, will surely result in exciting breakthroughs, not only for the treatment of FXS, but for a whole range of neurodevelopmental disorders.

## Data Availability

Data sharing is not applicable to this article as no new data were created or analysed.
